# Age-Related EEG Power Reductions Cannot Be Explained by Changes of the Conductivity Distribution in the Head Due to Brain Atrophy

**DOI:** 10.3389/fnagi.2021.632310

**Published:** 2021-02-18

**Authors:** Mingjian He, Feng Liu, Aapo Nummenmaa, Matti Hämäläinen, Bradford C. Dickerson, Patrick L. Purdon

**Affiliations:** ^1^Department of Anesthesia, Critical Care and Pain Medicine, Massachusetts General Hospital and Harvard Medical School, Boston, MA, United States; ^2^Harvard-MIT Health Sciences and Technology, Massachusetts Institute of Technology (MIT), Cambridge, MA, United States; ^3^School of Systems and Enterprises, Stevens Institute of Technology, Hoboken, NJ, United States; ^4^Athinoula A. Martinos Center for Biomedical Imaging, Massachusetts General Hospital and Harvard Medical School, Charlestown, MA, United States; ^5^Frontotemporal Disorders Unit, Department of Neurology, Massachusetts General Hospital and Harvard Medical School, Charlestown, MA, United States

**Keywords:** brain simulation model, cortical atrophy, Boundary Element Method, aging, EEG forward model

## Abstract

Electroencephalogram (EEG) power reductions in the aging brain have been described by numerous previous studies. However, the underlying mechanism for the observed brain signal power reduction remains unclear. One possible cause for reduced EEG signals in elderly subjects might be the increased distance from the primary neural electrical currents on the cortex to the scalp electrodes as the result of cortical atrophies. While brain shrinkage itself reflects age-related neurological changes, the effects of changes in the distribution of electrical conductivity are often not distinguished from altered neural activity when interpreting EEG power reductions. To address this ambiguity, we employed EEG forward models to investigate whether brain shrinkage is a major factor for the signal attenuation in the aging brain. We simulated brain shrinkage in spherical and realistic brain models and found that changes in the conductor geometry cannot fully account for the EEG power reductions even when the brain was shrunk to unrealistic sizes. Our results quantify the extent of power reductions from brain shrinkage and pave the way for more accurate inferences about deficient neural activity and circuit integrity based on EEG power reductions in the aging population.

## 1. Introduction

Decreases in scalp EEG amplitude have been described previously in normal aging (Polich, 1997) and in patients suffering from the Alzheimer's disease (AD) (Ehle and Johnson, 1977; Babiloni et al., 2006). These amplitude changes, often quantified as reductions in EEG power, have been interpreted to reflect disruptions in cortical activity, particularly in the alpha frequency band (Vlahou et al., 2014). The levels of power reductions can be quite large, even in putative normal aging. For example, a recent study examining EEG power during anesthesia showed that elderly subjects over the age of 80 can have age-related power reductions of up to 15 dB in the alpha (8–12 Hz) band compared to younger adults below the age of 30, corresponding to a ~6-fold change in amplitude (Purdon et al., 2015). Group comparisons of resting state alpha oscillations have shown power reductions up to 20 dB between healthy elderly and younger subjects (Breslau et al., 1989; Scally et al., 2018), with changes of a similar magnitude reported for P300 event-related potential responses (Polich, 1997). Relative to younger adults, power reductions between 3 and 5 dB have been observed in sleep EEG recordings in middle aged adults (Dijk et al., 1989; Carrier et al., 2001; Landolt and Borbély, 2001), which are further reduced by ~5 dB in elderly subjects above the age of 80 (Djonlagic et al., 2021). Significant changes in EEG signal amplitude are also common in neurological diseases. One study estimated power reductions in resting state alpha oscillations to be ~6 dB when comparing normal aging with mild cognitive impairment patients, which increased to ~10 dB for AD patients (Babiloni et al., 2013). Over the past decade, efforts have been made to derive aging and disease biomarkers from the EEG (Moretti et al., 2011; Babiloni et al., 2013; Miranda et al., 2019). This approach is appealing not only because of the empirical relationship between EEG amplitude and disease state, but also because the EEG contains information about underlying brain circuit dynamics and topography that might relate to disease mechanisms.

The striking reductions in EEG power during aging and AD could be explained by a number of factors. One interpretation would be that decreases in EEG power reflect underlying reductions in cortical current amplitudes due to reduced synaptic density, activity, synchronization, or some combination therein (Ishii et al., 2017). On the other hand, significant cortical atrophy can occur during aging, AD, and other age-related neurological diseases (Salat et al., 2004; Dickerson et al., 2009; Fjell et al., 2009; Thambisetty et al., 2010). Cortical atrophy is accompanied by reduced brain volume and an expansion in cerebrospinal fluid (CSF) volume to fill the space between the pia mater and the arachnoid mater. The CSF has a higher conductivity than both the brain and the skull, and it acts as a current shunt resulting in the attenuation of the electric potentials on the scalp, measured with EEG. Age-related EEG power reductions might therefore arise from additional attenuation of scalp potentials due to enlarged CSF space (Barzegaran et al., 2019).

It is important to gauge how much EEG power reductions can be attributed to purely anatomical changes of the conductor geometry, since the remaining power reductions would more accurately reflect neurological changes during aging. This distinction is also relevant when studying early AD patients, in whom both cortical atrophy and disrupted neural currents are present with much greater severity than those in normal aging. Given the growing interest in using EEG power reductions as potential biomarkers of aging and AD pathology (Moretti, 2016; Cassani et al., 2018; Toural et al., 2020; Turner et al., 2020; Paitel et al., 2021), a better understanding of the influence of altered conductor geometry is essential to interpret decreases in EEG power as evidence for neuronal changes during aging and disease.

In this study, we used MRI-based four-layer conductor models to quantify the extent to which EEG signal power reductions can be attributed to age-related brain structural changes. Specifically, our goal was to investigate whether increased distance between an atrophied cortical surface and scalp electrodes could explain the level of EEG signal attenuation experimentally observed in the aging population. We simulated this cortical shrinkage to compute resultant EEG signal changes, employing both spherical and realistic MRI-based brain models. The sensitivity of EEG signal amplitudes to varying tissue conductivities was also analyzed using these models. We found that physiologically plausible levels of cortical shrinkage mimicking the extremes of age-related diseases can only account for a modest fraction of the reduction in EEG power observed in previous studies. These results delineate the magnitude of signal attenuation due to cortical shrinkage and enable more accurate interpretations of age-related EEG power reductions.

## 2. Materials and Methods

### 2.1. Subject Data

Structural MRI scans were collected from one healthy young subject at Athinoula A. Martinos Center for Biomedical Imaging, Massachusetts General Hospital. The subject gave written informed consent, and the study protocol was approved by the Massachusetts General Hospital Human Research Committee. Brain structural anatomical details were obtained from a T1-weighted multi-echo sequence (MEMPRAGE, TR/TE/TI = 2,530/1.64/1,200 ms, 7° flip angle, 1.0 × 1.0 × 1.0 mm^3^ voxel resolution) and a fast low-angle shot gradient echo sequence (FLASH, TR/TE = 20/1.85 ms, 5° flip angle, 1.3 mm sagittal slice thickness, 1.3 × 1.0 mm^2^ in-plane resolution) using a 12-channel RF receive coil array on a 3T Siemens TrioTM MR scanner. An equidistant 128-channel montage (WaveguardTM, ANT Neuro, The Netherlands) was used for EEG electrode placement, and electrode positions were digitized using the Polhemus FastSCAN II laser scanner, aligned to the subject's structural MRI based on fiducial points (nasion and preauricular points) using the iterative closest point algorithm, and projected onto the scalp surface.

### 2.2. EEG Forward Models

We used forward models to quantify changes in the EEG signal that would occur under varying levels of cortical shrinkage. A forward model maps the cortical currents to electric potentials on scalp, measured in EEG (Sarvas, 1987; Mosher et al., 1999). Following well-established methods developed for EEG/MEG source localization and brain stimulation, we represented cortical currents as distributed current dipoles normal to the cortical mantle (Dale and Sereno, 1993) and calculated the corresponding scalp surface potentials using the Boundary Element Method (BEM) (De Munck, 1992; Gramfort et al., 2014). BEM formulates the bioelectric volume conduction problem using well-known surface integral equations that are solved in discretized form using triangulated boundary surfaces (Geselowitz, 1970; Horacek, 1973; Hamalainen and Sarvas, 1989). Many BEM modeling problems require only three layers: the skin, the skull, and the brain. In this work, an additional CSF layer was incorporated to capture the effect of altered conductivity distribution due to an expanded CSF compartment during aging.

We constructed both spherical and realistic MRI-based EEG volume conductor models. Current dipoles were fixed with orientations normal to the cortical surface, motivated by the understanding that the primary currents producing the EEG signals are believed to come from pyramidal cells aligned perpendicularly to the cortical surface (He et al., 2018). Four-layer spherical volume conductor models were built using the MNE-Python software (Gramfort et al., 2013) evaluated with spherical harmonic expansion (Berg and Scherg, 1994). Since scalp potentials in spherical models are solved with closed-form expressions, these models provide theoretical verification and generalization of the results in realistic models based on the single MRI scan. The layers are constituted of skin, skull, CSF, and brain with relative outer radii of 1.0, 0.90, 0.83, and 0.78. These radii were empirically derived from distances on the subject's T1 structural scan, using the average of distances measured in Freeview from the origin of the scan acquisition to the most anterior, posterior, and superior points of respective tissue types. A total of 2562 source dipoles were positioned on a sphere just beneath the brain sphere (0.99 times the brain radius). Based on existing literature describing the range of electrical conductivities for different head tissues (Mahdavi et al., 2018; Saturnino et al., 2019), conductivity values of 0.3, 0.006, 1.5, and 0.3 S/m were assigned to skin, skull, CSF, and brain tissues, respectively.

In the four-layer MRI-based realistic volume conductor models, we computed the electromagnetic field using the BEM solutions to approximate boundary surface potentials. Tissue segmentation and mesh construction of boundary surfaces were initialized by running two SimNIBS processing pipelines using default settings: (1) “mri2mesh” based on a FreeSurfer 6.0 reconstruction (Windhoff et al., 2013), and (2) “headreco” based on the CAT12 and SPM12 toolboxes (Nielsen et al., 2018). FLASH images were used in place of the T2-weighted images that are typically used in the SimNIBS pipeline to improve tissue segmentation. The initial pial surface output of “mri2mesh” was corrected for “over-grabbing” of skull regions on the brainmask as often described in FreeSurfer quality control pipelines (http://freesurfer.net/fswiki), and “mri2mesh” was repeated with this corrected pial surface to provide an improved, updated tissue segmentation.

**Figure 1:**
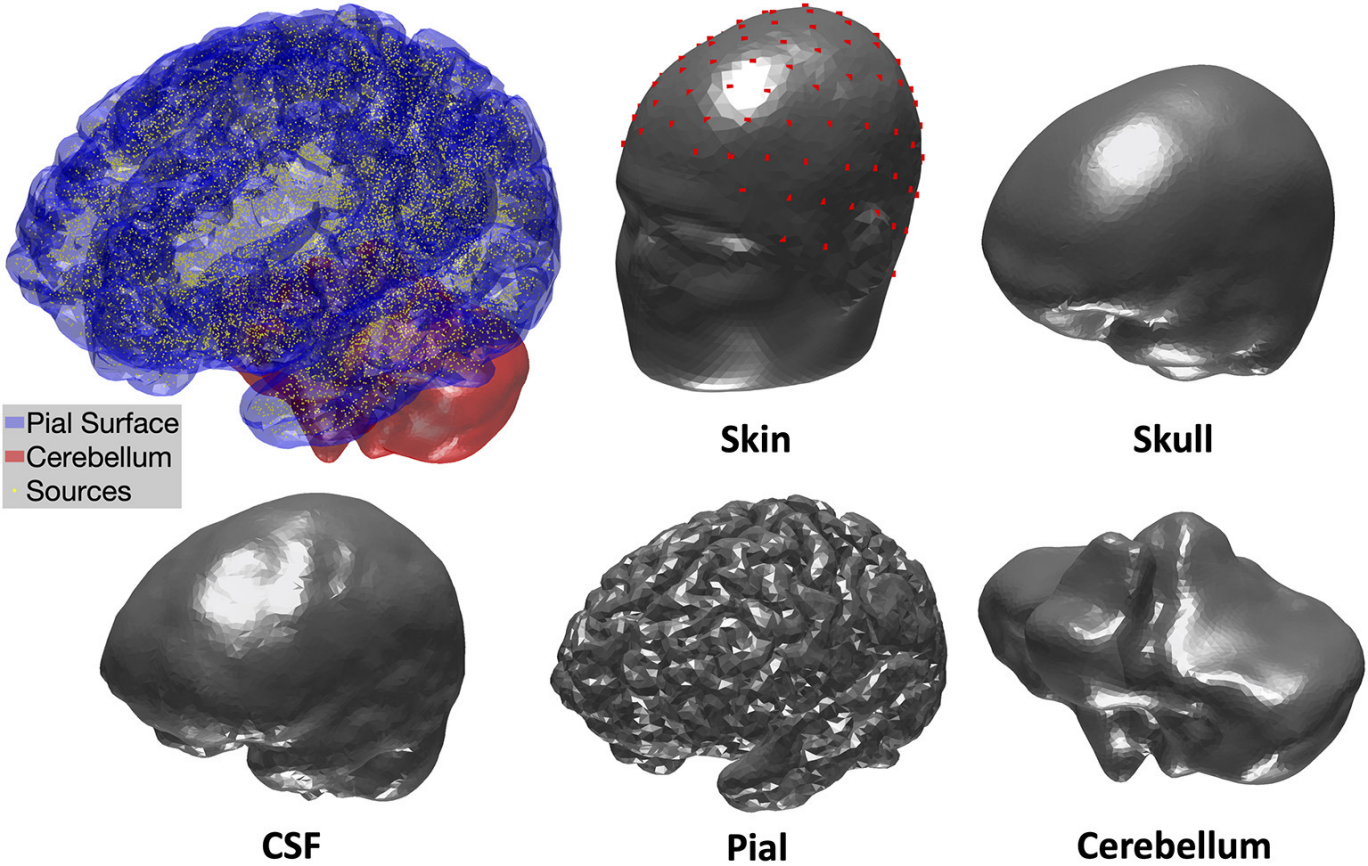
Visualization of cortical current dipole sources and tissue compartment BEM surfaces.

The gray matter/white matter boundary surface from “mri2mesh” was used to construct the source space in MNE-Python using a total of 20484 current dipole sources spanning the cortical surface. The pial, cerebellum, and inner skull (CSF) surfaces were obtained from “mri2mesh.” For the outer skull surface, CAT12 within the “headreco” pipeline provided more accurate segmentation of bone structures compared to “mri2mesh.” However, “headreco” outlined facial bone structures that were too detailed than appropriate for BEM solutions. Therefore, we constructed a hybrid outer skull surface combining the superior and posterior quadrants obtained with “headreco” and the anterior-inferior (facial) quadrant obtained using “mri2mesh” (see Supplementary Material for more details). Finally, the skin surface was extracted using the FreeSurfer watershed algorithm, which produced smoother surface estimates more appropriate for BEM modeling than “mri2mesh” or “headreco.” Figure 1 shows the final BEM surfaces. Additional details about the forward model, including smoothing parameters and vertex numbers, are included in the Supplementary Material. We assigned skin, skull, CSF, and brain tissues with conductivity values of 0.3, 0.006, 1.5, and 0.3 S/m, respectively, just as in the spherical models. The compartments inside the cerebellum and pial surfaces were both labeled as brain tissue.

Forward model gain matrices for the realistic models were obtained by solving the four-layer BEM problem. A direct non-iterative solver can be applied to the standard BEM formulation although this limits the resolution of the volume conductor model. Approaches utilizing the Fast Multipole Method (FMM) acceleration have been developed (Kybic et al., 2005) for high-resolution models but iterative numerical solvers are needed (Makarov et al., 2021). Here, the standard BEM formulation allows modeling CSF with adequate spatial resolution, and we selected a validated and efficient linear collocation BEM solver using the isolated source approach (Stenroos and Nummenmaa, 2016).

### 2.3. Simulation of Cortical Shrinkage

**Figure 2:**
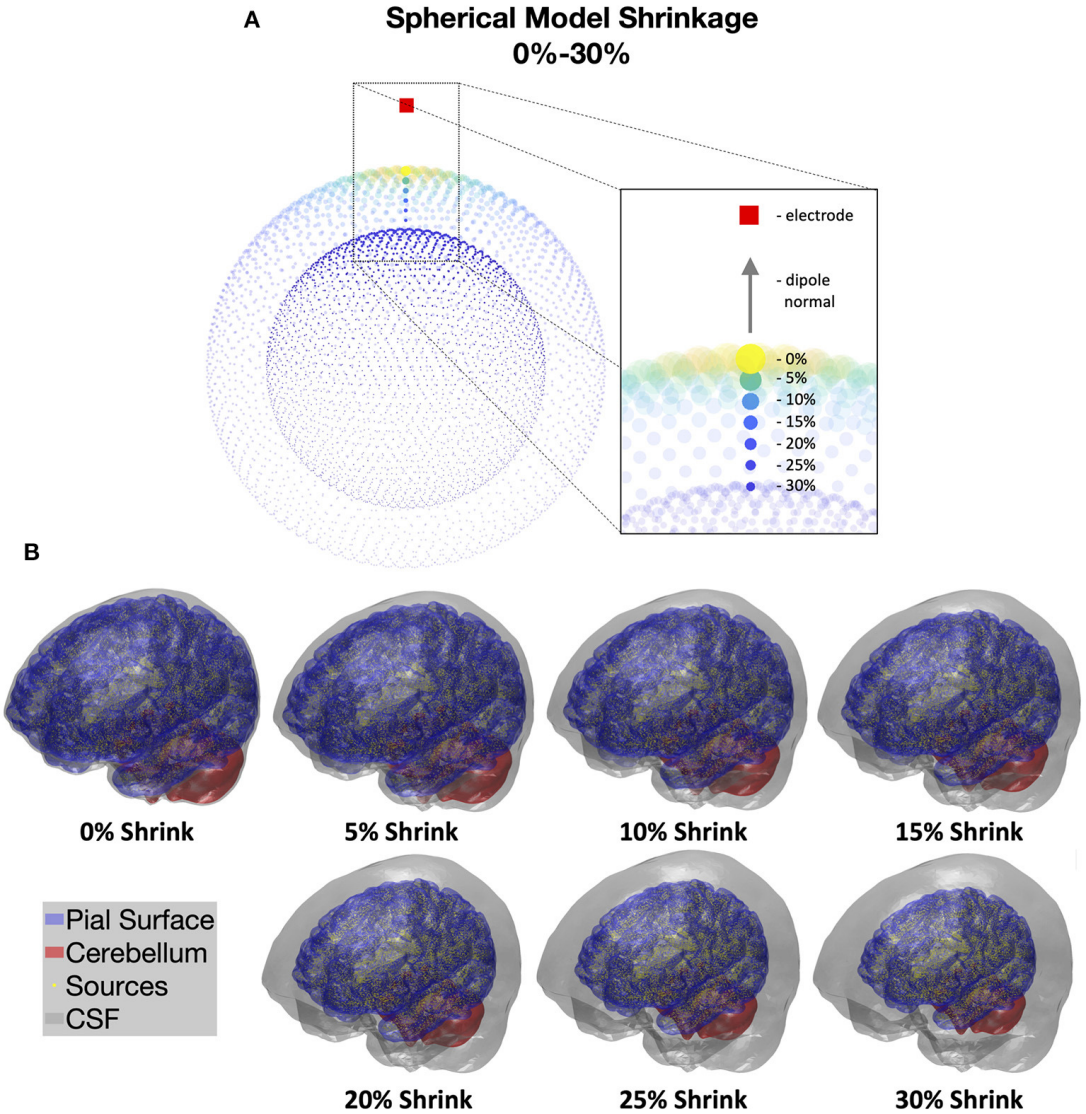
**(A)** Illustration of cortical shrinkage simulations in the spherical models through 0–30% shrinkage values. Brighter color and larger dot sizes correspond to larger gain values in the forward model gain matrices. Dipole orientations are kept the same during cortical shrinking and normal to the underlying surfaces. **(B)** Cortical shrinkage simulations in the realistic BEM models through 0–30% shrinkage values. CSF volumes are shown in gray, which expand with increased cortical shrinking; pial surfaces are in blue; cerebellum surfaces are in red; and cortical current dipole sources are shown in yellow. Estimates of CSF thickness in the current models are: 0% = 2.9 mm, 5% = 5.8 mm, 10% = 8.9 mm, 15% = 12.0 mm, 20% = 15.3 mm, 25% = 18.6 mm, 30% = 22.0 mm.

We simulated the overall structural effect of brain atrophy by shrinking the radius of the brain while maintaining the dimensions of the other tissue layers, i.e., skull and scalp. This “brain shrinkage” moves cortical dipoles away from the scalp and expands the CSF layer, in a manner analogous to age-related brain atrophy. This whole-brain shrinkage was implemented by reducing the distance from each source to the median centroid of all source dipoles by varying percentages of 0-30% in increments of 5% in both the spherical and realistic MRI-based models. We maintained the same number of dipoles across all levels of shrinking. For the spherical models, due to symmetry, only a single electrode was needed to characterize power reductions measured on the scalp (Figure 2A). To simulate the EEG signal changes, a patch of 20 source dipoles (~3.5 cm^2^) closest to the scalp EEG electrode was activated, while keeping the other sources silent. Each dipole in the active patch was assumed to have the same amplitude. The power reduction for each shrinkage value was calculated directly from the forward model gain matrices (see Supplementary Material for details).

In the realistic models, simulated shrinkage affected different cortical regions slightly differently due to anatomical variations and non-spherical geometry. The pial surface and the dipole locations were shrunk over the same range of percentages, 0-30% in 5% increments, to model the expansion of the CSF layer under cortical atrophy (Figure 2B). We estimated the corresponding average CSF thickness for each shrinkage value by computing the mean nodal projection distance from the CSF surface vertices to the pial/cerebellum surfaces (0% = 2.9 mm, 5% = 5.8 mm, 10% = 8.9 mm, 15% = 12.0 mm, 20% = 15.3 mm, 25% = 18.6 mm, 30% = 22.0 mm). CSF thickness can be estimated to be between 3-6 mm in normal aging (Pfefferbaum, 1990; Salat et al., 2004), while severe AD patients have been reported to have CSF thicknesses of up to 10 mm (Ancora et al., 2018). Thus, cortical shrinkage values of up to 10% (8.9mm) are within a physiologically plausible range, but global shrinkage above 10% would be implausible. Power reductions were computed at each of 128 electrodes to adequately sample EEG signals across the whole scalp. Similar to the approach for the spherical models, for each EEG electrode position, we activated the top 20 source dipoles with the highest squared gain magnitudes and estimated the power reduction over a local neighborhood of 4 to 7 electrodes. This procedure was repeated for each electrode position to estimate the average overall power reduction. We show a 2D topological plot of the electrode montage and neighboring electrodes in the Supplementary Material (Supplementary Figure 1). All active dipoles were assumed to have the same amplitude. Therefore, power reductions were calculated directly from the forward model gain matrices (see Supplementary Material for details). The final power reduction for each shrinkage value was represented using the mean and standard deviation of dB differences across all 128 electrodes.

### 2.4. Conductivity Analyses

As described earlier, we set the tissue conductivities in our models to values at the midpoints of the published ranges. However, multiple studies have highlighted how uncertainties in skull and CSF conductivities can impact on the accuracy of forward models (Gençer and Acar, 2004; Stenroos and Nummenmaa, 2016; Saturnino et al., 2019; Vorwerk et al., 2019; Koulouri and Rimpilainen, 2020). In addition, several studies have noted that tissue conductivities can change with advancing age (Wendel et al., 2010; Moskalenko et al., 2011; Mohammed et al., 2017; Thomas et al., 2018). To better understand the sensitivity of EEG power reductions to variations in CSF and skull conductivities, we repeated the cortical shrinkage simulations employing the full ranges of reported CSF and skull conductivity values (CSF: 1.2–1.8 S/m in 100 steps, skull: 0.003–0.015 S/m in 100 steps). The conductivities were varied in a spherical model with a fixed shrinkage percentage of 10%, which corresponds to a mean CSF thickness of 8.9 mm in the realistic models, approximating the largest physiologically plausible shrinkage encountered in aging. Spherical models instead of realistic models were used for this analysis to increase generalizability of the results and to decrease computation time in sampling the conductivity space with high resolution.

## 3. Results

### 3.1. Cortical Shrinkage in Spherical Models

**Figure 3:**
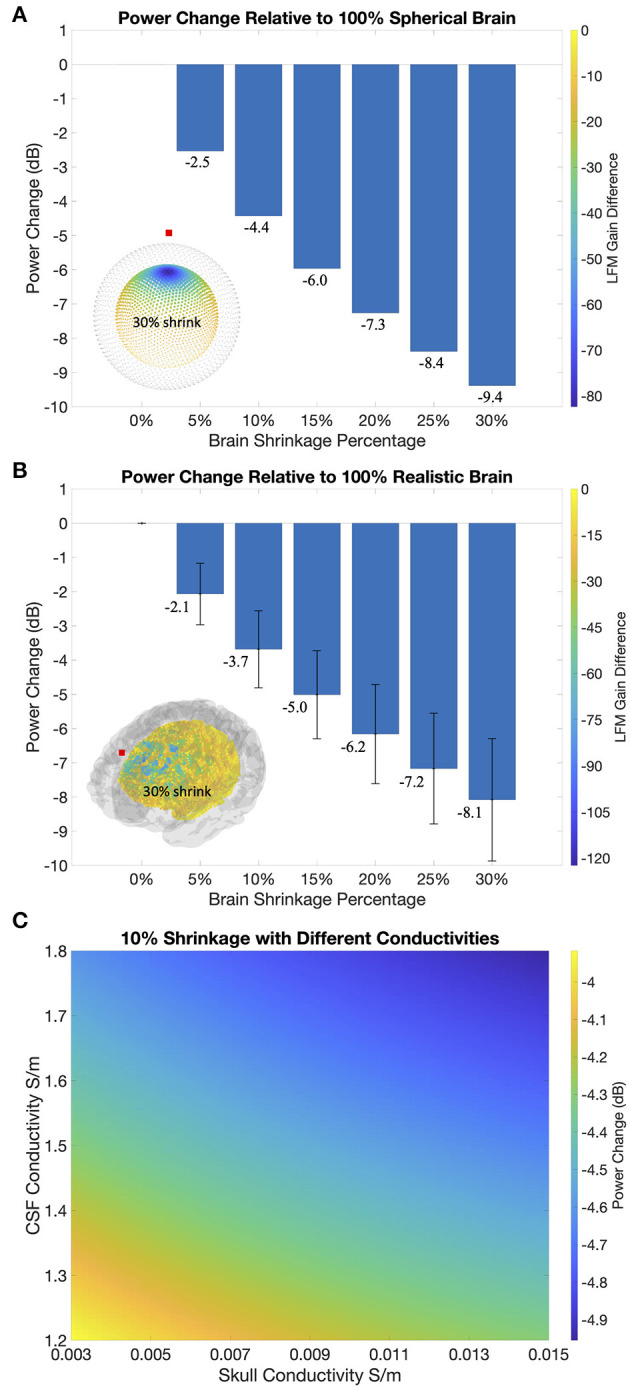
**(A)** Power reductions in the spherical models. Bottom left corner shows absolute gain differences in the forward models between intact source space (gray) with the 30% shrunk source space (colored). Cooler color and larger dot sizes represent greater gain differences. LFM = lead field matrix = forward model gain matrix. **(B)** Power reductions in the realistic BEM models. Bottom left corner shows absolute gain difference between intact (gray) and 30% shrunk (colored) source spaces. Error bars correspond to standard deviations. **(C)** Variations of power reductions in a 100 pixel x 100 pixel grid as CSF conductivity varies between 1.2 and 1.8 S/m and skull conductivity varies between 0.003 and 0.015 S/m.

Figure 3A shows the EEG power reductions observed in spherical models at different levels of cortical shrinkage. At the unrealistically high 30% level of shrinkage, we observed a power reduction of less than 10 dB compared to the original spherical volume conductor model. At more physiologically plausible shrinkage values of 5 and 10%, simulated power reductions were 2.5 and 4.4 dB, respectively. In Figure 3A, we also illustrate the difference in the lead field matrix (LFM) absolute gain for a given electrode at 30% shrinkage compared to no shrinkage. The highest attenuation of measured source activity occurred directly below the measured electrode, as one would predict from principles of volume conduction. This observation corroborates our decision to activate the 20 dipole sources nearest to the electrode (see Supplementary Figure 2), since doing so provides an approximate upper bound on the power reductions that could be expected from a given level of shrinkage.

### 3.2. Cortical Shrinkage in Realistic Models

Figure 3B shows the EEG power reductions (mean and standard deviation) across different levels of brain shrinkage, averaged across all active source patches and electrode positions. Power reduction values in these realistic models were similar to those observed under the spherical models. At the highest level of shrinkage (30%, mean CSF thickness = 22.0 mm) simulations produced less than 10-dB power reductions compared to the intact model with no shrinkage. Meanwhile, at physiologically plausible 5% and 10% levels of shrinkage, we obtained power reductions of 2.1 dB (std = 0.9) and 3.7 dB (std = 1.1), respectively. In Figure 3B, we plot the difference in the LFM absolute gain for a frontal electrode at 30% shrinkage compared to no shrinkage. Similar to the spherical models, gain magnitudes decreased with increasing distance from the electrode, albeit with variations that reflect the changing dipole orientations along cortical folds. Since the quasistatic approximation of Maxwell's equations applies in EEG at physiological frequencies <100 Hz, the shrinkage-dependent attenuation we calculate here is frequency independent in the frequency range of interest (Supplementary Figure 3).

### 3.3. Sensitivity of EEG Attenuation to Tissue Conductivity Values

Figure 3C shows, for a fixed cortical shrinkage of 10%, how the EEG attenuation changes as a function of varying CSF and skull conductivities. This analysis shows that changes in conductivity across wide ranges can alter the EEG attenuation by at most 0.5 dB. EEG signal attenuation is therefore relatively insensitive to uncertainties in tissue conductivity. This result is compatible with previous findings of the sensitivity of EEG amplitudes to tissue conductivities (Stenroos and Nummenmaa, 2016), as the power reduction quantified here in the dB scale takes the ratio between shrinkage values, which normalizes out absolute changes on EEG amplitudes.

## 4. Discussion

In this study we analyzed the extent to which age-dependent EEG signal power reductions could be attributed to structural changes in the brain. Using both spherical and realistic MRI-based forward models, we found that physiologically plausible levels of cortical atrophy produced only modest decreases in EEG power, which are small compared to those seen in aging and age-related dementia (Babiloni et al., 2013; Purdon et al., 2015). In addition, the EEG attenuation was insensitive to uncertainties in CSF and skull tissue conductivity values. These results show that although brain structural changes can reduce the size of the EEG signal via altered conductor geometry during volume conduction, the structural changes cannot account for the full extent of EEG signal decreases reported during aging and dementia. It follows that the remaining reduction in EEG power would be primarily attributable to underlying neurophysiological changes.

A critical component of our approach was to use four-layer BEM forward models to provide anatomically accurate simulations of shrinking cerebral volume that could incorporate the corresponding increase in CSF volume. This approach requires accurate extraction of tissue boundary meshes from MRI scans. We devised a novel workflow combining three different MRI mesh-extraction pipelines in FreeSurfer, SimNIBS, and MNE-Python to take advantage of the best properties of these respective methods. We also analyzed four-layer spherical models and observed EEG power reductions that were highly comparable to those observed using the realistic models. Corroborating results from the spherical and realistic models together provided consistent estimates of EEG power reductions from structural brain atrophy in aging. Potential age-related changes in the skull and skin thickness were not evaluated in this study after careful consideration. The skull thickness does not change appreciably with advancing age beyond early adulthood except in cases of specific pathology (Albert et al., 2007). The skin thickness can change in varying ways during aging: it can increase as a result of obesity, or become thinner during cachexia, both of which are common during aging. Thus we deemed the known age-related decrease in cerebral volume and the accompanying increase in CSF thickness as the most relevant variables in our models.

**Figure 4:**
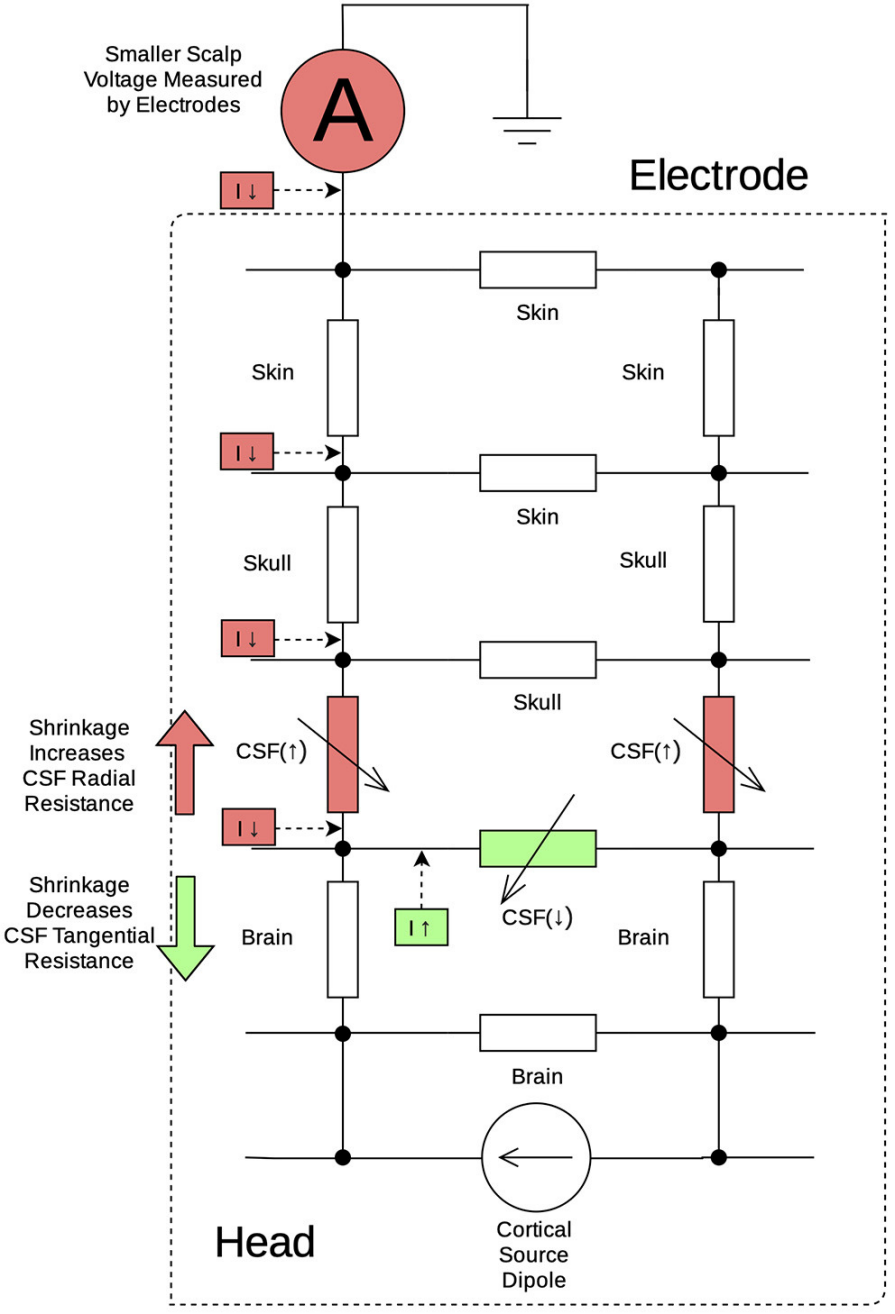
Equivalent circuit diagram illustrating changes to CSF resistance during cortical shrinkage: as the CSF layer expands, there is increased CSF resistance (red resistors) in the radial direction (red arrow) leading to decreased current conducting toward the scalp electrode (red boxes). At the same time, there is decreased CSF resistance (green resistor) in the tangential direction (green arrow) leading to increased current shunting away from the scalp electrode and flowing back to cortical current dipoles.

Our simulations modeled cerebral atrophy as a “shrinkage” in the brain parenchymal volume while holding other tissue boundaries constant. This simulation procedure captures three different effects of real brain atrophy. First, a primary effect of the shrinkage was to increase the thickness of the CSF layer. As shown in Figure 4, this expanded CSF space attenuates the EEG signal in two ways: first by increasing the CSF radial resistance and reducing currents in the interior-exterior direction (in Red), and second by decreasing CSF tangential resistance to shunt currents away from the scalp electrodes (in Green). Second, cortical source dipoles were pulled away from the scalp electrodes, increasing the distance of volume conduction and consequently signal attenuation. Third, as the pia mater was shrunk along with the cortex, the distance from the cortical gray/white matter boundary surface to the pial surface also decreased, capturing cortical thinning in brain atrophy. The reduced distance from current dipoles to the closest boundary surface (brain/CSF) also contributed to greater cancellation of electrical signals from the source. This effect can be explained using a secondary electric field created by charge accumulation along the boundary surface (Geselowitz, 1967). All three effects above are purely due to alterations of the conductor geometry, given that we maintained the same dipole strength across shrinkage percentages. Crucially, the BEM models incorporate these effects at once, allowing us to model the overall EEG power reductions from structural changes of brain atrophy concisely represented by a single parameter of CSF thickness.

In our simulations, the CSF layer expanded by more than three-fold from a starting point of 2.9 mm average thickness at 0% shrinkage, to 5.8 and 8.9 mm at 5 and 10% shrinkage, respectively. The latter value is comparable to the CSF thicknesses observed in severe AD (Ancora et al., 2018). Despite these large changes in CSF thickness, the EEG signal attenuation was modest: 2.1 dB for 5% shrinkage and 3.7 dB for 10% shrinkage in the realistic models. Larger, physiologically implausible levels of shrinkage were required to reduce EEG power to levels approaching the ~10 dB decrements observed in aging and age-related neurological disease.

An important caveat of the simulation analyses is that the inter-subject variability of anatomical details including the extent of cortical atrophy is not modeled. Our approach combines simulated shrinkage using realistic models based on a single MRI and generalizable spherical models with results that are highly consistent. Power reductions are quantified in this study similar to a longitudinal study design, while empirically observed age-related EEG power changes are cross-sectional comparisons with individual differences contributing to the level of power reductions observed. Additional factors such as cortical folding and scalp location when measuring EEG signals can also influence the absolute scales of power reductions (Supplementary Figure 3). Our simulations activated the top 20 dipoles with the highest signal strength to quantify a relative upper bound on power reductions. In addition, spherical models provide theoretical estimates on power reductions expected to result from shrinkage. Hence, our results could be interpreted to represent average changes we would expect to see when comparing groups of aging or dementia patients with younger adults. Comparisons of any two particular brains could differ from these results due to inter-subject variability. Future studies could address the question of inter-subject variability by analyzing imaging datasets of aging and AD subjects using the BEM modeling approach presented here.

Aside from the modest influence on volume conduction investigated here, age-related decreases in EEG power can also reflect an overall reduction in the size of cerebral currents. This could occur through a variety of mechanisms, including reductions in post-synaptic current amplitude, neuronal density, and/or neuronal synchrony, all of which have significant functional and pathophysiologic implications. Overall, head anatomy varies substantially between individuals and across age ranges. Realistic MRI-based forward models could be employed to gauge the individualized impact of structural changes on scalp EEG signals, to facilitate accurate interpretations of experimental findings of EEG power reductions, and to maximize the diagnostic precision of EEG-based biomarkers for age-related neurological disorders. Our results suggest that while structural changes due to brain atrophy play a role, age-dependent attenuation of the EEG signal more likely reflects underlying functional and neurophysiological changes that are fundamental characteristics of aging and age-related disease.

## Data Availability Statement

The raw data supporting the conclusions of this article will be made available by the authors upon request, without undue reservation.

## Ethics Statement

The studies involving human participants were reviewed and approved by Massachusetts General Hospital/Brigham and Women's Hospital Institutional Review Board. The patients/participants provided their written informed consent to participate in this study.

## Author Contributions

MH: formal analysis, software, data curation, and first draft writing. FL: data curation, formal analysis, software, and review-editing. AN: software, supervision, and review-editing. MH: review-editing and supervision. BD: review-editing. PP: grant acquisition, supervision, conception and review-editing. All authors contributed to the article and approved the submitted version.

## Conflict of Interest

The authors declare that the research was conducted in the absence of any commercial or financial relationships that could be construed as a potential conflict of interest.
