# Supplementary material for: Age-Related EEG Power Reductions Cannot Be Explained by Changes of the Conductivity Distribution in the Head Due to Brain Atrophy
Source: Front Aging Neurosci. 2021 Feb 18;13:632310. doi: 10.3389/fnagi.2021.632310 (PMC7929986; doi:10.3389/fnagi.2021.632310)
Supplement: Supplementary file 1 [file Data_Sheet_1.PDF]

Supplementary Material

1 REALISTIC BEM MODEL BOUNDARY MESHES

As detailed in the method section, the BEM boundary surfaces for the realistic models were derived from different tissue segmentation pipelines. For the outer skull surface, due to the advantages of CAT12 segmentation of bone structures, “headreco” resulted in a far more accurate outer skull surface than “mri2mesh” but also contained additional facial bone structures that were too detailed and thin for BEM solutions. Hence, a hybrid outer skull surface was created using customized MATLAB functions to morph “mri2mesh” outer skull surface into “headreco” outer skull surface in the superior and posterior quadrants while maintaining the original “mri2mesh” outer skull surface in the anterior-inferior portion (anterior to foramen magnum and inferior to eyebrows) to avoid introducing additional facial bone structures. Specifically, a horizontal plane was defined using the most anterior and posterior points of the ”mri2mesh” surface followed by a vertical plane defined posterior to the spinal cord from the ”headreco” surface. Then in the upper three quadrants (excluding the quadrant containing the facial structures), the ”mri2mesh” surface vertices were radially dilated towards the ”headreco” surface and then projected to the closest points (not necessarily vertices) on the ”headreco” surface to update new vertex positions. The vertex positions of the ”mri2mesh” surface in the anterior quadrant were kept unchanged. This resulted in a hybrid skull surface that aligned with ”mri2mesh” in the anterior quadrant and aligned with the ”headreco” surface in the other three quadrants, which were the most important for EEG signal volume conduction.

All resultant dense boundary meshes (skin, skull, CSF, pial, cerebellum) were slightly smoothed using the smoothsurf function in iso2mesh toolbox, downsampled with equal-geodesic-distant vertices using the remeshTriSurfDistMap function in GIBBON toolbox, corrected for topological errors with meshfix toolbox, and checked for proper nesting ordering (skin $>$ skull $>$ CSF $>$ pial = cerebellum $>$ sources), no intersection, and complete watertightness. Smoothing parameters and vertex numbers are shown below in Table S1. Aligned electrodes were then projected onto the skin surface. Lastly, source dipoles that were too close to the smoothed pial surface were shifted away in the opposite projection direction to ensure minimal cortical thickness of 0.5mm while maintaining dipole orientations to ensure numerical stability in field computations.

Table S1. BEM surface smoothing and geodesic-distance-based downsampling parameters.

BEM surfaces	Skin	Skull	CSF	Pial	Cerebellum
**Pipeline**	MNE-Python	SimNIBS mri2mesh + headreco	SimNIBS mri2mesh	SimNIBS mri2mesh	SimNIBS mri2mesh
**Smooth iterations**	0	200	200	100	50
**Vertex Number**	4096	8188	8185	16162	8191

Although our choice of 0.5mm minimal cortical thickness was narrower than the 1.5mm distance recommended for the Helsinki BEM solver (Stenroos and Nummenmaa (2016)), we were using a

fine vertex resolution for the pial surface (16162 vertices compared to a typical choice of 8000+ vertices) due to the needs of shrinkage simulations. In addition, erroneous pial regions had been corrected after the initial “mri2mesh” run using FreeSurfer. Visual inspections of the gain matrices confirmed reasonable scaling for all sources in the forward models.

2 POWER REDUCTION CALCULATIONS

EEG forward modeling takes the following form, where $\mathbf{x}$ represents scalp electrode potentials; G represents the forward model gain matrix; and $\mathbf{s}$ represents a vector of source dipole activities. Given the assumption of identical source activities across the n activated dipoles, $\mathbf{s}$ is formed by n identical non-zero entries and 0 elsewhere, and in the current models $n = 20$.

$$\mathbf{x} = \mathbf{G} \cdot \mathbf{s}$$

$$\mathbf{s} = (0 \ldots s_1 \ldots s_n \ldots 0)^T$$

Let s represent a time-series of length N in the cortical source space for a single current dipole, which is the same as all activated dipoles $\{s_1, s_2, \ldots, s_{20}\}$, then the scalp electrode time-series x_k that is of length N and measured at the kth electrode can be expressed as a linear combination of activities from different dipoles scaled by different gains in the forward model:

$$x_k = \sum_{i=1}^{20} \big(G_{k,c_i} \times s_i\big) = s \sum_{i=1}^{20} G_{k,c_i}$$

where G_{k,c_i} is the entry of G at row k and column c_i; c_i is the column number for the ith activated dipole in the forward model gain matrix G. EEG signal power is then estimated from x_k. If we take the multitaper spectral estimation method (Prerau et al. 2017) with L tapers on the time-series $x_k = (x_{k,1}, x_{k,2}, \ldots, x_{k,t=N})$, the spectral power estimate at any frequency f is:

$$\hat{S}(f) = \frac{1}{L} \sum_{l=1}^{L} \left| \sum_{t=1}^{N} w_t^l x_{k,t} e^{2\pi k f \Delta t} \right|^2$$

Cortical shrinkage simulations generate different forward model gain matrices $\tilde{G}$ that capture altered volume conduction. Assuming identical source dipole activities across shrinkage values, we arrive at a different time-series $\tilde{x}_k$ under a particular cortical shrinkage value. The spectral power of $\tilde{x}_k$ is estimated accordingly:

$$\tilde{x}_k = s \sum_{i=1}^{20} \tilde{G}_{k,c_i}$$

$$\hat{S}_{\text{shrink}}(f) = \frac{1}{L} \sum_{l=1}^{L} \left| \sum_{t=1}^{N} w_t^l \tilde{x}_{k,t} e^{2\pi k f \Delta t} \right|^2$$

Power reductions ΔP in the dB scale are calculated with the following equation regardless of frequency f since the EEG forward model is a linear time-invariant system:

$$
\begin{aligned}
\Delta P &= 10\log_{10}\hat{S}_{\text{shrink}}(f) - 10\log_{10}\hat{S}(f) \\
&= 10\log_{10}\frac{\hat{S}_{\text{shrink}}(f)}{\hat{S}(f)} \\
&= 10\log_{10}\frac{\frac{1}{L}\sum_{l=1}^{L}\left|\sum_{t=1}^{N} w_t^l \tilde{x}_{k,t} e^{2\pi k f \Delta t}\right|^2}{\frac{1}{L}\sum_{l=1}^{L}\left|\sum_{t=1}^{N} w_t^l x_{k,t} e^{2\pi k f \Delta t}\right|^2} \\
&= 10\log_{10}\frac{\frac{1}{L}\sum_{l=1}^{L}\left|\sum_{t=1}^{N} w_t^l \left(s_t \sum_{i=1}^{20}\tilde{G}_{k,c_i}\right) e^{2\pi k f \Delta t}\right|^2}{\frac{1}{L}\sum_{l=1}^{L}\left|\sum_{t=1}^{N} w_t^l \left(s_t \sum_{i=1}^{20} G_{k,c_i}\right) e^{2\pi k f \Delta t}\right|^2} \\
&= 10\log_{10}\frac{\left(\sum_{i=1}^{20}\tilde{G}_{k,c_i}\right)^2 \frac{1}{L}\sum_{l=1}^{L}\left|\sum_{t=1}^{N} w_t^l s_t e^{2\pi k f \Delta t}\right|^2}{\left(\sum_{i=1}^{20} G_{k,c_i}\right)^2 \frac{1}{L}\sum_{l=1}^{L}\left|\sum_{t=1}^{N} w_t^l s_t e^{2\pi k f \Delta t}\right|^2} \\
&= 10\log_{10}\left(\frac{\sum_{i=1}^{20}\tilde{G}_{k,c_i}}{\sum_{i=1}^{20} G_{k,c_i}}\right)^2
\end{aligned}
$$

As can be seen from the last equality, power reductions can be computed directly without spectral estimation by taking the squared ratio of sums of gain values and converting to the dB scale.

3 EEG ELECTRODE MONTAGE

A topological plot of the equidistant 128-channel montage (Waveguard^{TM}, ANT Neuro, The Netherlands) is shown in Figure S1.

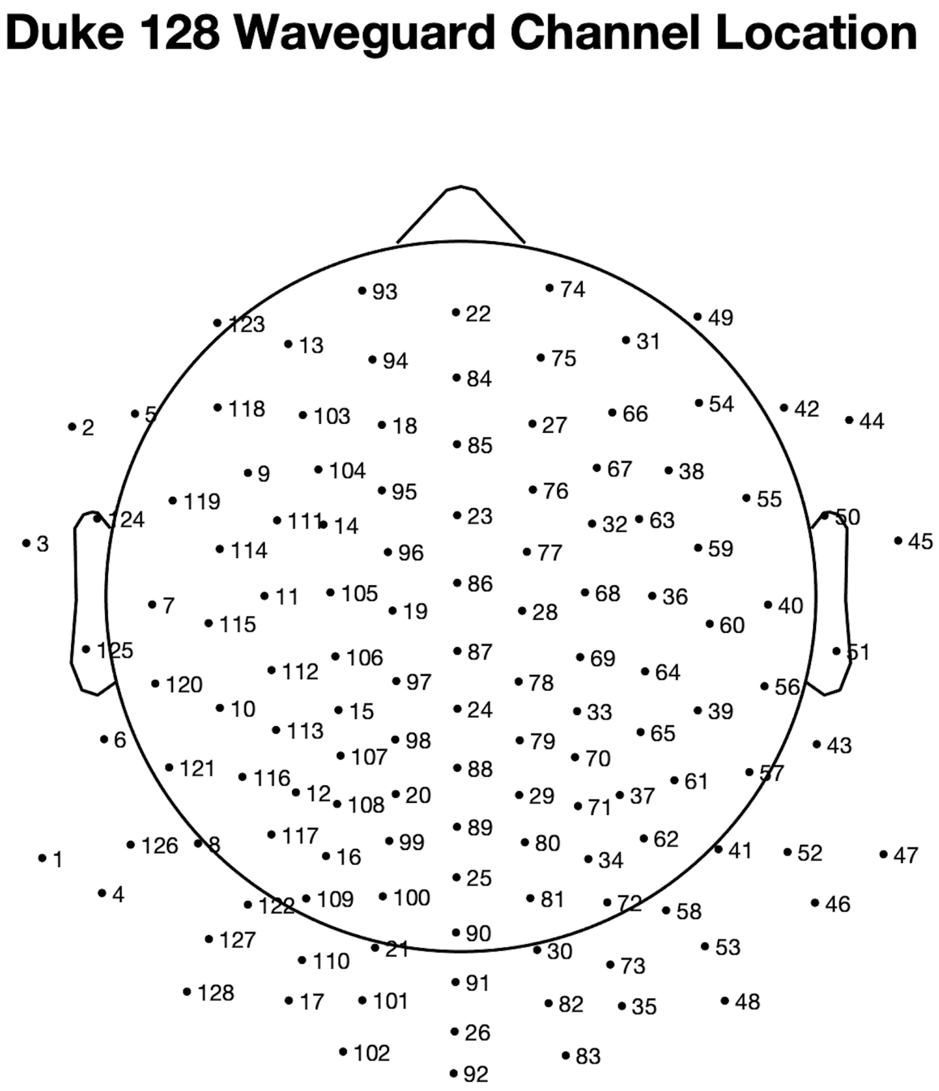

Figure S1. Channel locations of the equidistant 128-channel montage.

4 20 ACTIVATED SOURCE DIPOLES

In both spherical and realistic models, we activated 20 source dipoles with the highest squared gain magnitudes for a scalp electrode to estimate a loose upper bound on power reductions. 20 source dipoles that were activated in the spherical models are shown in Figure S2 for both 30% shrinkage and intact volume conductor models. As can be observed from the overlaid gain difference visualization, the 20 source dipoles (outlined with magenta color) with the highest squared gain magnitudes were the same dipoles undergoing the most gain attenuation during cortical shrinking. Hence, by activating these source dipoles with high gain values, our estimates of the power reductions were higher than the power reductions obtained if we activated all sources or some distant sources with lower gain values.

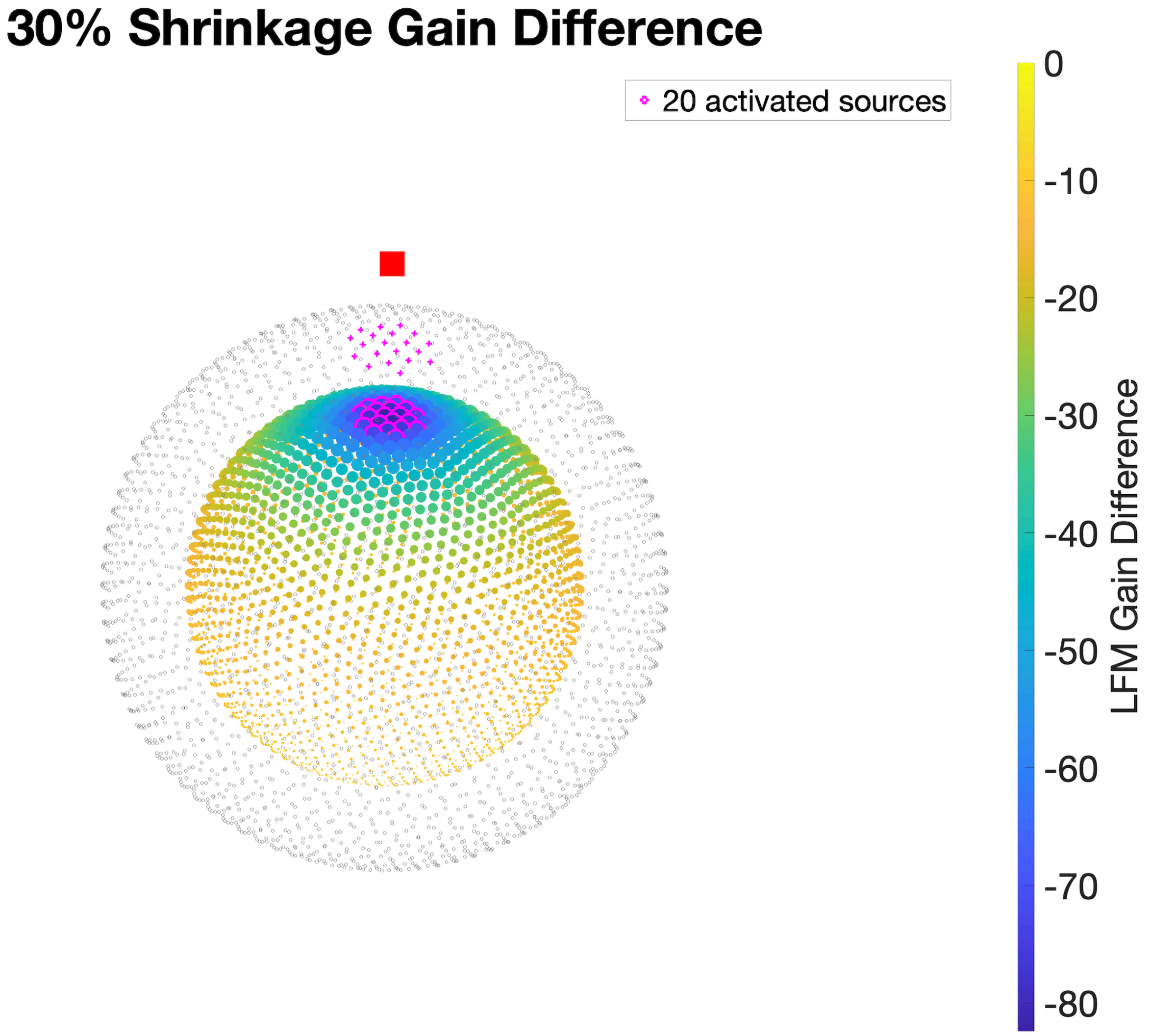

Figure S2. Activated 20 source dipoles with the highest squared gain magnitudes underwent the most gain attenuation during cortical shrinkage simulations. Gray dots represent source dipoles in the intact source space. Colored and size-weighted dots represent source dipoles in the 30% shrinkage model, with larger dot size and cooler color corresponding to greater gain attenuations in the forward model gain matrices. The 20 activated sources were outlined in magenta for both intact and 30% shrinkage source spaces. LFM = lead field matrix = forward model gain matrix.

5 CORTICAL ATROPHY SIMULATION SPECTRA FOR DIFFERENT ROIS IN THE REALISTIC MODELS

As derived earlier in the Supplementary material, power reduction calculations were the same between directly using forward model gain matrices and propagating source activities to scalp electrodes followed by spectral estimations. We found it helpful to provide some realistic spectra for EEG electrode potentials during cortical shrinkage simulations. We selected five ROIs often investigated in EEG studies (rostral middle frontal, superior frontal, superior parietal, inferior parietal, and lateral occipital) and estimated spectral powers measured by an electrode (and its neighbors) immediately above each of five ROIs. ROIs were identified on the cortical surface using the Desikan-Killiany-atlas (Desikan et al. (2006)) segmentation in FreeSurfer, and we activated 20 source dipoles in each ROI using a real intracranial recording. The intracranial recording was a short 30s segment with 100Hz sampling frequency shown in Figure S3A from a patient under deep anesthesia. Written informed consent was obtained from the subject, and the experimental procedures were approved by the Massachusetts General Hospital/Brigham and Women's Hospital Institutional Review Board. After propagating the activities through EEG forward model gain matrices, spectral estimation was done using the multitaper method (Prerau et al. (2017)) using a window size of 5s with 1s step size, time-half-bandwidth product of 2 and 3 tapers, 2^{16} discrete Fourier transform points (nfft), and linear detrending within each window. Mean spectra shown in Figure S3B-F were computed by taking the mean of spectral estimates from the 6 windows during the 30s. It should be stressed that these spectra were presented only as an alternative way to visualize power reductions using a more familiar format of spectrum at magnitudes consistent with real power spectra observed in experimental studies. The simulated power reduction values were exactly the same as summarized by the bar plots (Figure 3A&B) in the main text.

As can be observed from the spectra plots, the absolute power magnitudes were different across the five ROIs due to different anatomical details and volume conduction distances to scalp electrodes. The power reductions in the dB scale were fairly similar across ROIs with only small variations at the 5% shrinkage level. It's worth pointing out that the spectrum shapes were identical, since the same intracranial recording time-series was used as the source activity across shrinkage values. The power reductions were also identical across different frequencies. In real experimental data, selective reduction was often observed in a frequency band such as in the alpha range. This can be reconciled considering regional specific cortical atrophies producing certain oscillations rather than global shrinkage as simulated in the current models. Detailed analyses of regional shrinkage were omitted from this study. The proper method to analyze regional-specific and frequency-specific power reductions is by building realistic 4-layer forward models based on MRIs from different individuals across age ranges and simulating source activities based on a prior knowledge of the location of certain oscillatory activities (such as eyes-closed occipital alpha).

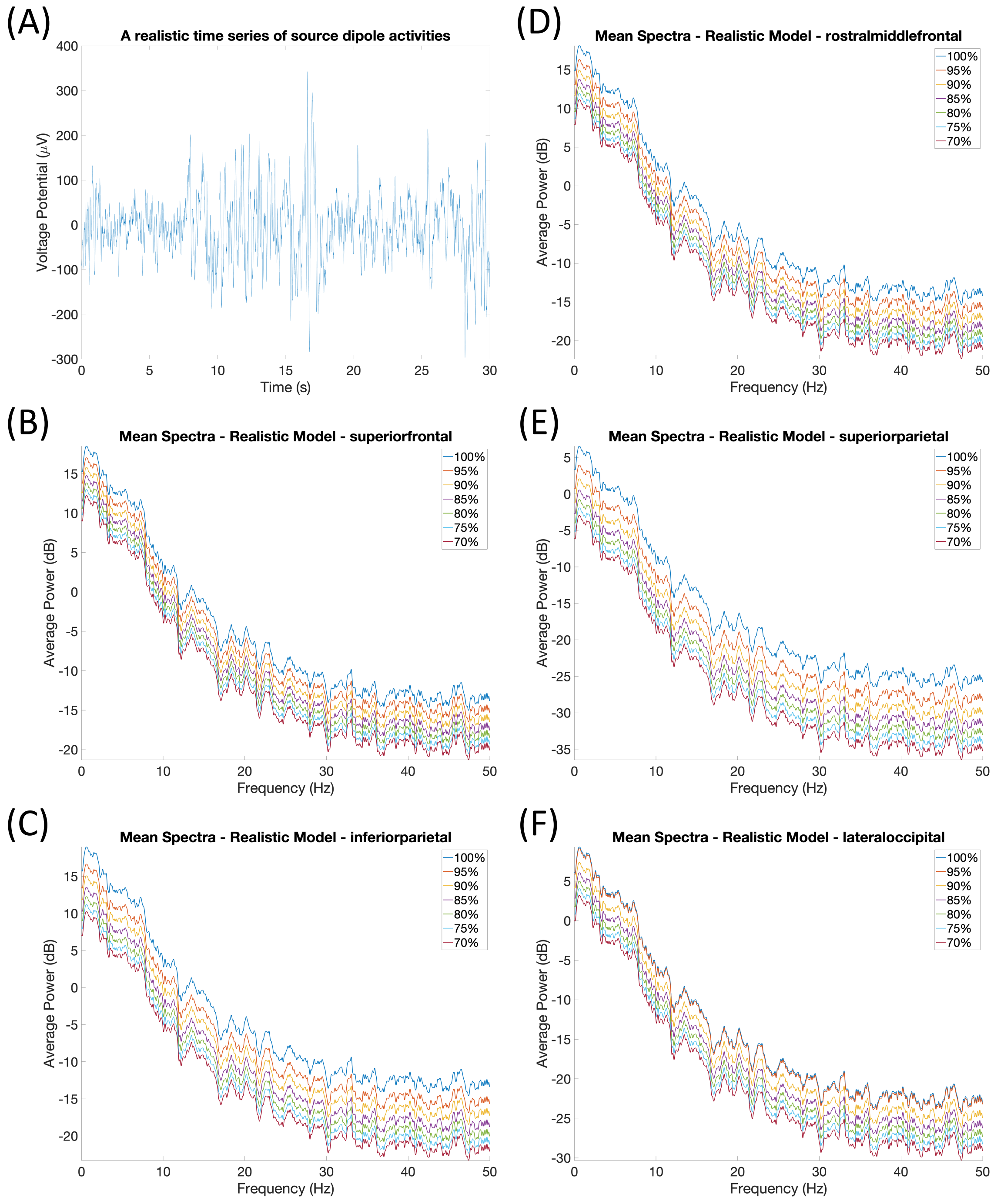

Figure S3. (A) 30s time-series of an intracranial recording used as source activity. Details of the recording and spectral estimation parameters are described in text of Section 5 in the Supplementary Material. **(B)** Mean spectra averaged over 6 time windows (5s each) for a set of 7 electrodes above the superior frontal lobes with 20 dipoles activated using the signal shown in **(A)**, **(C)** Mean spectra for the inferior parietal lobes, **(D)** Mean spectra for the rostral middle frontal lobes, **(E)** Mean spectra for the superior parietal lobes, **(F)** Mean spectra for the lateral occipital lobes. Colored lines in **(B)-(F)** correspond to different levels of cortical shrinkage percentages as labelled in the legends.

REFERENCES

Desikan, R. S., Ségonne, F., Fischl, B., Quinn, B. T., Dickerson, B. C., Blacker, D., et al. (2006). An automated labeling system for subdividing the human cerebral cortex on mri scans into gyral based regions of interest. *Neuroimage* 31, 968–980

Prerau, M. J., Brown, R. E., Bianchi, M. T., Ellenbogen, J. M., and Purdon, P. L. (2017). Sleep neurophysiological dynamics through the lens of multitaper spectral analysis. *Physiology* 32, 60–92

Stenroos, M. and Nummenmaa, A. (2016). Incorporating and compensating cerebrospinal fluid in surface-based forward models of magneto-and electroencephalography. *PLoS One* 11
